# 

**Published:** 1878-08

**Authors:** 


*CONTENTS.*

| | Page |
|---|---|
| Teeth | 543 |
| Hereditary Disease | 547 |
| True Courage | 548 |
| Economy a Duty | 549 |
| A Sweet Philosophy | 554 |
| How to Admomish | 555 |
| Prejudice | 555 |
| How Ladies Should Dress | 556 |
| Ingratitude to Parents | 557 |
| Entering a Room | 558 |
| A Good Woman Never Grows Old | 558 |
| Flowers for Winter | 559 |
| The Nature of Consumption | 559 |
| Pickles | 560 |
| Five Minutes' Value | 560 |
| Life Uncertain | 560 |
| How to Save a Drowning Person | 561 |
| Ice Water | 562 |
| Regimen | 563 |
| Sleeping | 564 |
| Tobacco Users | 566 |
| Cellars | 567 |
| Small Pox | 568 |
| To Keep Apples | 568 |

